# Inhibition and enhancement of contextual fear memory destabilization

**DOI:** 10.3389/fnbeh.2014.00144

**Published:** 2014-04-28

**Authors:** Jonathan L. C. Lee, Charlotte R. Flavell

**Affiliations:** School of Psychology, University of BirminghamEdgbaston, Birmingham, UK

**Keywords:** memory reconsolidation, boundary conditions, reactivation, CB1 receptor

## Abstract

The reactivation of a memory can result in its destabilization, necessitating a process of memory reconsolidation to maintain its persistence. Here we show that the destabilization of a contextual fear memory is potentiated by the cannabinoid CB1 receptor agonist Arachidonyl-2-chloroethylamide (ACEA). Co-infusion of ACEA and the IkappaB kinase (IKK) inhibitor sulfasalazine (Sulf) into the dorsal hippocampus impaired contextual fear memory reconsolidation. This observation was achieved under behavioral conditions that, by themselves, did not result in a reconsolidation impairment by Sulf alone. Moreover, we show that the destabilization of a contextual fear memory is dependent upon neuronal activity in the dorsal hippocampus, but not memory expression *per se*. The effect on contextual fear memory destabilization of intra-hippocampal ACEA was replicated by systemic injections, allowing an amnestic effect of MK-801. These results indicate that memory expression and destabilization, while being independent from one another, are both dependent upon memory reactivation. Moreover, memory destabilization can be enhanced pharmacologically, which may be of therapeutic potential.

## Introduction

Memory reconsolidation describes the process that is disrupted when an amnestic treatment is administered at the time of memory reminder, leading to long-lasting memory impairment (Lewis, 1979; Nader and Hardt, 2009). Thus the reconsolidation process is required to restabilize a memory that has been destabilized following a reminder (Nader et al., 2000). Memory reactivation, defined as the hypothesized reactivation by a reminder of the memory trace that is necessary for retrieval and behavioral expression, does not, however, necessarily lead to memory destabilization and reconsolidation. There are a number of boundary conditions that describe the parametric conditions under which a reminder fails to trigger reconsolidation, as evidenced by a lack of observed amnestic effect (see Nader and Hardt, 2009, for review). In relation to contextual fear memories, stronger and older memories appear to be more difficult to destabilize, requiring more extensive re-exposure to the context in order to successfully trigger memory reconsolidation (Suzuki et al., 2004).

A limited number of mechanisms have been delineated that are essential for memory destabilization. First revealed was the necessity for NR2B-containing NMDA receptors (NMDARs) in the basolateral amygdala for the destabilization of auditory fear memories (Ben Mamou et al., 2006). However, contextual fear memories are notable for being the setting for the fullest exploration of the mechanisms of memory destabilization, with a requirement for synaptic protein degradation (Lee et al., 2008), AMPA receptor subunit endocytosis (Rao-Ruiz et al., 2011), L-type voltage-gated calcium channels (Suzuki et al., 2008) and cannabinoid CB1 receptors (CB1R; Suzuki et al., 2008).

There are also a growing number of studies showing that memory destabilization does not require successful memory expression. First the mechanisms of destabilization are doubly dissociable from those that are essential for memory expression (Ben Mamou et al., 2006). Moreover, impairment of memory expression did not prevent that memory from destabilizing in rodent taste aversion (Rodriguez-Ortiz et al., 2012) and object recognition (Balderas et al., 2013) memories, nor in an invertebrate contextual fear memory in the crab (Barreiro et al., 2013), although it has not previously been shown to apply to contextual fear memories. However, it is well established, and indeed a formal requirement, that memory destabilization and reconsolidation depend upon the reactivation of the memory trace (Dudai, 2004; Nader and Hardt, 2009). This, therefore, raises a distinction between reactivation, expression and destabilization, as partially noted by Barreiro et al. (2013). Therefore, it may be possible pharmacologically to distinguish between memory reactivation, destabilization and expression.

In the present study, we tested the hypotheses both that memory destabilization can be enhanced pharmacologically to engage memory reconsolidation, and that memory destabilization is dependent upon memory reactivation-related neural activity, rather than memory expression *per se*. We tested these hypotheses in a contextual fear memory setting, in which there are established mechanisms of memory destabilization, reconsolidation and expression. In particular, we targeted hippocampal CB1Rs through direct intrahippocampal infusions or systemic injections to stimulate memory destabilization. The efficacy of memory destabilization was assessed through the presence of an amnestic effect of the IkappaB kinase (IKK) inhibitor sulfasalazine (Sulf), which regulates the activity of the transcription factor nuclear factor-kappa B and has previously been demonstrated to impair the reconsolidation of contextual fear memories (Lubin and Sweatt, 2007; Lee and Hynds, 2013). Moreover, we used intra-hippocampal infusions of the sodium channel blocker TTX to disrupt neuronal activity, as well as the metabotropic glutamate receptor group 1 agonist 3HPG, both of which have been demonstrated to impair the expression of contextual fear memories (Lorenzini et al., 1996; Szapiro et al., 2001).

## Materials and Methods

### Subjects and Surgical Procedures

Sixty three adult male Lister Hooded Rats (Charles River, UK), weighing 275–325 g at the time of surgery, were implanted with chronic indwelling cannulae targeting the dorsal hippocampi as described previously (Lee et al., 2004). A further 80 adult male Lister Hooded rats, weighing 200–225 g at the start of the experiment, were used for the systemic drug administration experiments. All procedures were conducted in compliance with the United Kingdom Animals (Scientific Procedures) Act 1986 (PPLs 40/3205 and 70/7662). At the end of the experiment, the rats were killed by rising concentration of CO_2_ and, for the cannulated rats, their brains freshly extracted. For histological analysis, brains were stored in 4% paraformaldehyde for at least 7 days. The brains were subsequently sectioned at 60 μm and stained with Cresyl Violet to confirm cannula placements.

### Infusions and injections

Infusion procedures were carried out as previously described (Lee, 2008). Rats were habituated to the infusion or injection procedure on a single occasion prior to the start of behavioral procedures. All infusions were carried out in a volume of 1 μl per hemisphere at a rate of 0.5 μl/min. The final concentrations of drugs used were as follows:

- Sulf (Sigma-Aldrich, UK)—2 μg/μl (in sterile PBS with 10 mM HEPES plus 20% DMSO) (Lee and Hynds, 2013)
- ACEA (Arachidonyl-2-chloroethylamide; Abcam, UK)—5 pg/μl (in sterile PBS with 0.1% DMSO) (Clarke et al., 2008)
- 3HPG ((*S*)-3-Hydroxyphenylglycine; Tocris, UK)—33 pg/μl (in sterile PBS) (Szapiro et al., 2001)
- TTX (Tetrodotoxin; Abcam, UK)—10 ng/ μl (in sterile 0.1 mM citrate buffered saline) (Lorenzini et al., 1996)

**Figure 1:**
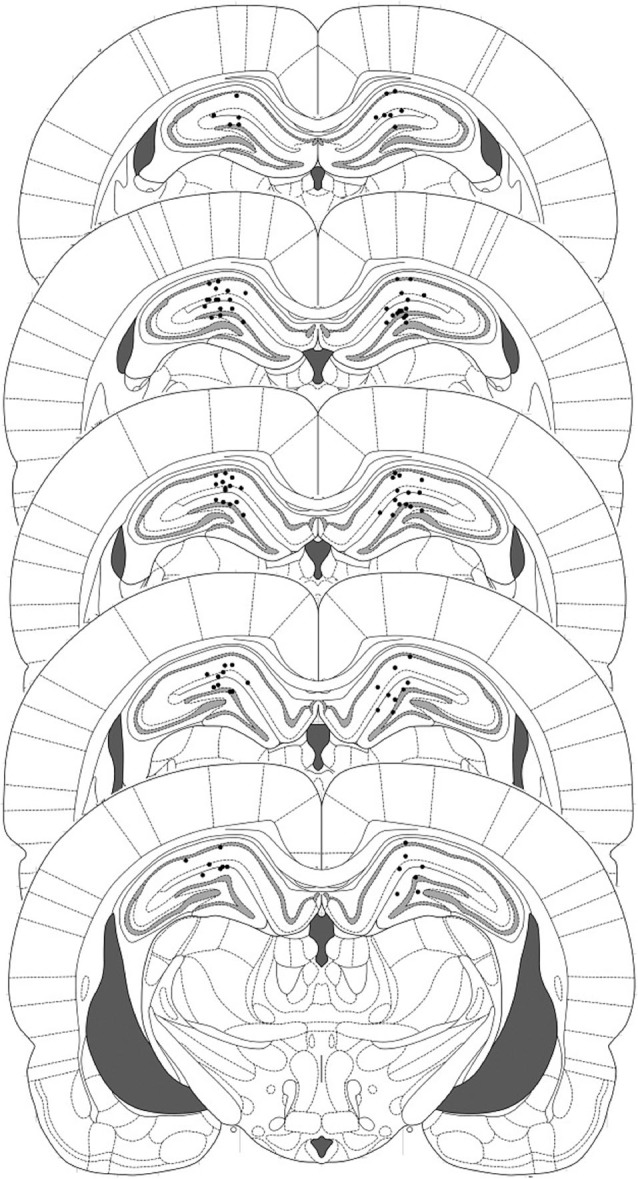
**Representation of injector placements in the dorsal hippocampus**. Histological evidence of injector tips was observed and correspond to the filled circles.

**Figure 2:**
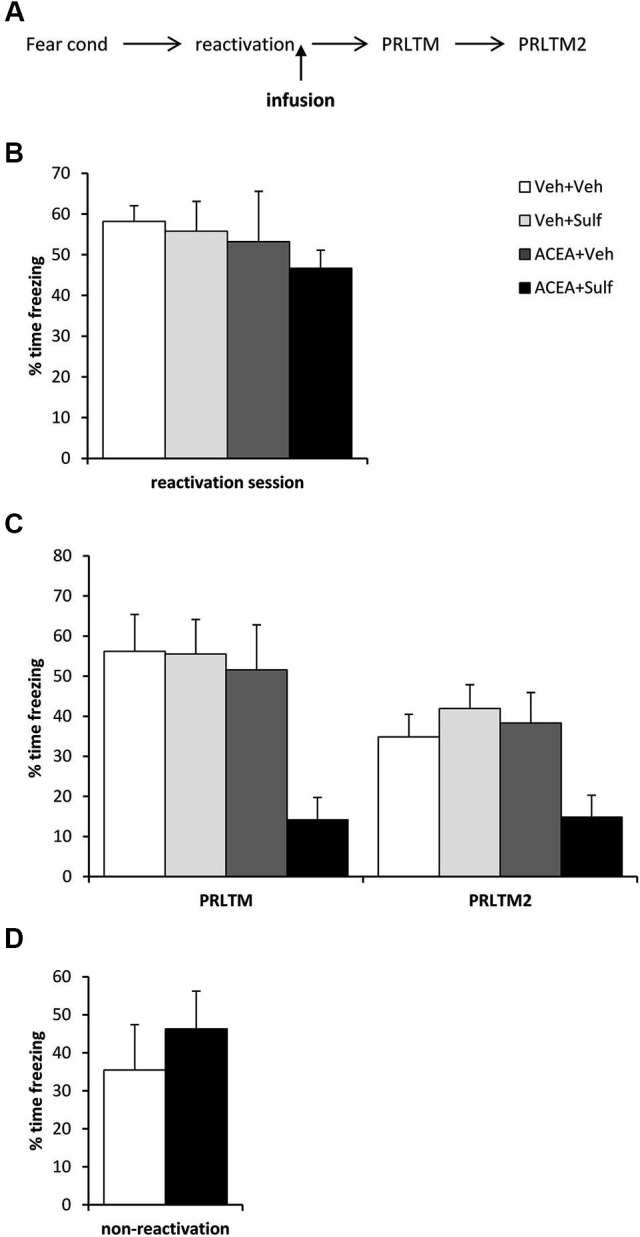
**Stimulation of memory destabilization by CB1 receptor agonism**. (**A**) Schematic representation of the experimental procedure. (**B**) Prior to infusion, there were no pre-existing differences in contextual freezing at memory reactivation. (**C**) Co-infusion of ACEA and sulfasalazine (Sulf) into the dorsal hippocampus immediately after memory reactivation impaired memory reconsolidation, whereas infusion of ACEA or Sulf alone had no amnestic effect. Contextual freezing was assessed 24 h (post-reactivation long-term memory, PRLTM) and 7 days (PRLTM2) after memory reactivation. (**D**) Co-infusion of ACEA and Sulf in the absence of memory reactivation did not affect subsequent contextual freezing. Data presented as mean + s.e.m. (*N* = 6–7 per group).

For the data in Figure 1, stock solutions of double the final concentration of Sulf and ACEA were prepared and then mixed with equal volumes of each other (for co-infusion of Sulf and ACEA) or the alternative vehicle (for infusions of Sulf or ACEA alone). The vehicle control consisted of equal volumes of the two vehicles for Sulf and ACEA. For the data in Figure 2, the vehicle group consisted of equal numbers of rats receiving the 3HPG and TTX vehicles. There were no obvious differences between the two vehicles in the behavior of the rats. For the systemic drug experiments, ACEA (5 mg/kg in 40:30:30 ethanol:DMSO:saline), SR141716A (0.2 mg/kg in 10% DMSO and 0.1% Tween-80 in saline) and MK-801 (0.1 mg/kg in saline) were injected intraperitoneally.

### Behavioral Procedures

Training and testing took place in four operant chambers as described previously (Lee and Hynds, 2013). For conditioning, rats were placed individually into the chambers. The houselight was illuminated and, after 2 min, the rats were subjected to three unsignaled 2-s, 0.5-mA footshocks, with an inter-trial interval of 2 min. The rats were left for 1 min following the final footshock before the houselight was extinguished and the rats were removed from the chambers. Twenty four hours later, the rats were returned to the context for a 2-min reactivation session. They were infused immediately after the session (Figure 2A; Sulf and/or ACEA), and/or 10 min prior to the session (3HPG or TTX). Injections took place 30 min before (SR141716A), 5 min before (ACEA) and immediately after (MK-801) the reactivation session. Rats were randomly allocated to experimental groups. A further 24 h later, the rats were returned to the context for a 2-min test (post-reactivation long-term memory, PRLTM). A final test took place 7 days after memory reactivation (PRLTM2). Behavior was video-recorded throughout the reactivation and test sessions and automatically scored for freezing using Videotrack software (Viewpoint Life Sciences, France).

### Statistical Analyses

The freezing data (time spent freezing) were converted into a percentage of time spent freezing during each session and analyzed in SPSS. The data were checked for normality and sphericity. As all data conformed to assumptions of normality and sphericity, no corrections were necessary. The test sessions were analyzed together in multifactorial mixed ANOVAs with factors Test, Sulf, ACEA and Group as appropriate. For the experiments involving Sulf + MK-801 and ACEA, the two drugs were separated onto different factors, resulting in a 3-way ANOVA. Significant interactions were explored using 2-way mixed ANOVAs to assess simple effects for the local infusion study. Given these observed effects, planned comparisons were conducted for the systemic injection studies. For the experiment involving 3HPG and TTX or SR141716A, there was a single Drug factor that was further explored with Sidak-corrected *post-hoc* tests. All analyses were conducted with alpha set at 0.05.

## Results

### Intra-hippocampal ACEA + sulfasalazine impairs post-reactivation contextual freezing

Eleven rats were excluded from the statistical analysis due to cannula misplacement or failure to complete the full schedule of testing (through ill health or experimenter error). The remaining operated rats had cannulae located bilaterally in the dorsal hippocampus (Figure 1). After contextual fear conditioning, there were no differences between the groups in the levels of contextual freezing at the reactivation session (Figure 2B; ACEA × Sulf interaction: (*F*_(1,21)_ = 0.073, *p* = 0.79); main effect of ACEA: (*F*_(1,21)_ = 0.84, *p* = 0.37); main effect of Sulf: (*F*_(1,21)_ = 0.34, *p* = 0.56)). At the subsequent test sessions (Figure 2C), there was a significant ACEA × Sulf interaction (*F*_(1,21)_ = 7.91, *p* = 0.01), with no session × ACEA × Sulf interaction (*F*_(1,21)_ = 0.14, *p* = 0.71). Analysis of simple main effects revealed no effect of Sulf alone (main effect of Sulf: *F*_(1,10)_ = 0.22, *p* = 0.65; session × Sulf: *F*_(1,10)_ = 0.31, *p* = 0.59), but a significant effect of Sulf in the ACEA-infused groups (main effect of Sulf: *F*_(1,11)_ = 10.03, *p* = 0.01; session × Sulf: *F*_(1,11)_ = 2.12, *p* = 0.17). Moreover, there was no effect of ACEA in isolation (main effect of ACEA: *F*_(1,11)_ = 0.004, *p* = 0.95; session × ACEA: *F*_(1,11)_ = 0.32, *p* = 0.59). Thus the co-infusion of ACEA and Sulf, but neither infusion alone, impaired subsequent contextual freezing.

In order both to rule out the possibility that the combined infusion of ACEA and Sulf had non-specific effects on memory, and to confirm that the observed impairment was dependent upon memory reactivation, we co-infused ACEA and Sulf in the absence of memory reactivation (Figure 2D). Compared to vehicle control, ACEA and Sulf did not impair subsequent contextual freezing (*F*_(1,10)_ = 0.48, *p* = 0.50). This demonstrated the reactivation-dependence of the ACEA + Sulf-mediated amnesia.

### Intra-hippocampal TTX, but not 3HPG protects against post-reactivation contextual freezing impairments

**Figure 3:**
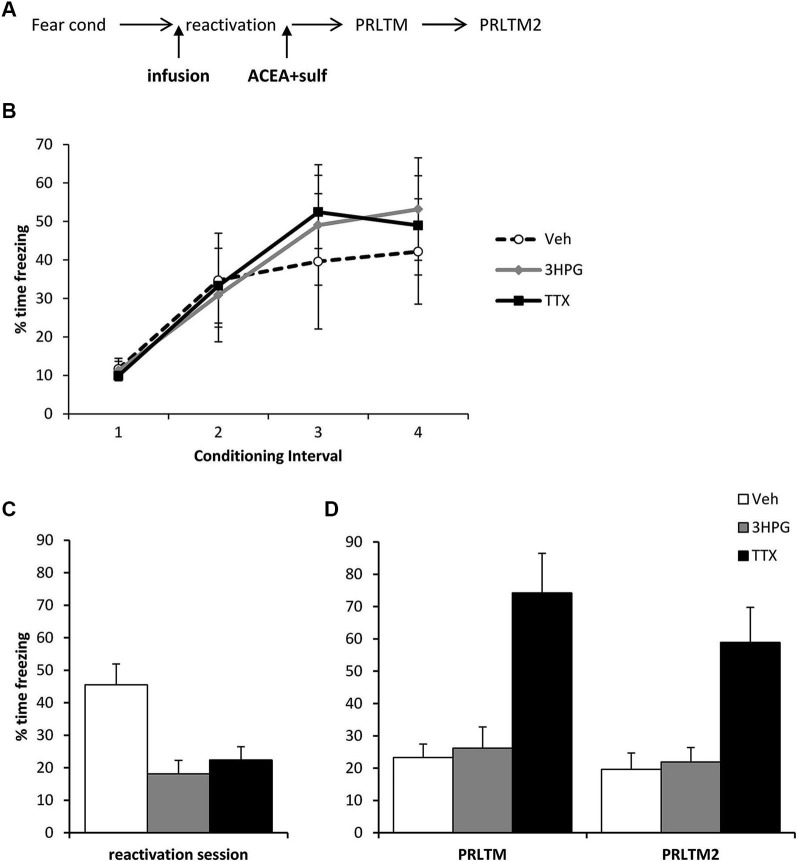
**Prevention of memory destabilization by TTX, but not 3HPG**. (**A**)**** Schematic representation of the experimental procedure. Rats were infused with TTX or HPG prior to memory reactivation, and then ACEA + Sulf immediately after the reactivation session. (**B**)**** There were no differences in contextual freezing at conditioning during the pre-shock (1), inter-shock (2 and 3) and post-final shock (4) intervals. (**C**)**** Intra dorsal hippocampal infusions of**** 3HPG and TTX impaired the expression of contextual fear at memory reactivation. (**D**)**** TTX impaired contextual fear memory destabilization. Contextual freezing was assessed 24 h (PRLTM) and 7 days (PRLTM2) after memory reactivation. TTX-infused rats froze more than 3HPG- and vehicle-infused rats at both tests. Data presented as mean + s.e.m. (*N* = 5 per group).

The amnestic effect of the ACEA + Sulf co-infusion was not dependent upon successful expression of the memory, although it did require neuronal activity in the dorsal hippocampus during memory reactivation. Three groups of rats were all infused with ACEA and Sulf immediately after memory reactivation, using the same behavioral parameters as before. The groups received additional infusions of vehicle, 3HPG or TTX 10 min prior to memory reactivation (Figure 3A). While there were no differences between the groups during fear conditioning (Figure 3B; Group × Interval: *F* = 0.44, *p* = 0.85; Group: *F*_(2,12)_ = 0.08, *p* = 0.93), both 3HPG and TTX acutely impaired the expression of the contextual fear memory (Figure 3C; *F*_(2,12)_ = 10.87, *p* = 0.002). *Post-hoc* analyses (*p* < 0.05) confirmed that both TTX and 3HPG acutely reduced freezing. At the post-reactivation tests (Figure 3D), the groups infused with vehicle and 3HPG showed low levels of contextual freezing, comparable to that observed previously in Figure 2C. In contrast, the rats infused with TTX showed high levels of contextual freezing. Overall analysis of the post-reactivation tests revealed a main effect of Drug (*F*_(2,12)_ = 14.80, *p* = 0.001) with no session × Drug interaction (*F*_(2,12)_ = 0.007, *p* = 0.99). *Post-hoc* analyses (*p* < 0.05) confirmed that TTX-infused rats froze more across both tests than the other groups.

**Figure 4:**
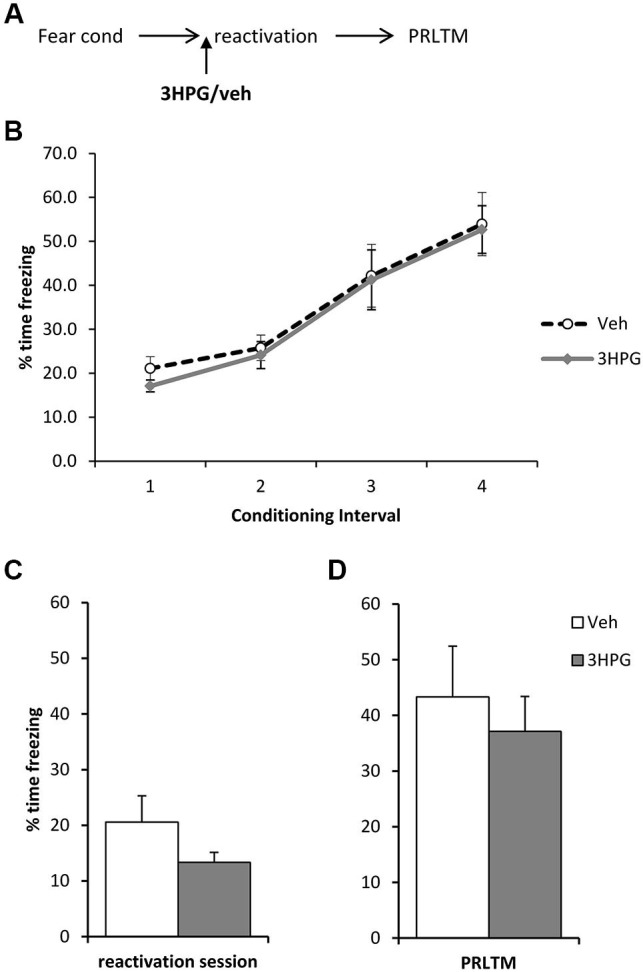
**No effect of pre-reactivation 3HPG alone**. (**A**) Schematic representation of the experimental procedure. (**B**) There were no differences in contextual freezing at conditioning during the pre-shock (1), inter-shock (2 and 3) and post-final shock (4) intervals. (**C**) Intra dorsal hippocampal infusions of**** 3HPG did not affect the expression of contextual fear at memory reactivation. (**D**) 3HPG had no effect on contextual fear memory 24 h (PRLTM) after memory reactivation. Data presented as mean + s.e.m. (*N* = 8 per group).

In order to rule out the possibility that 3HPG impaired reconsolidation independently of the infusion of ACEA and Sulf, we infused 3HPG (or vehicle control) prior to memory reactivation, with no post-reactivation infusions (Figure 4A). Again, there were no differences between the groups during fear conditioning (Figure 4B; Group × Interval: *F*_(3,42)_ = 0.06, *p* = 0.98; Group: *F*_(1,14)_ = 0.18, *p* = 0.68). There was no statistically significant effect of 3HPG at the reactivation session (Figure 4C; *F*_(1,14)_ = 2.38, *p* = 0.15), with the levels of freezing at reactivation in the vehicle group notably lower than previously observed in Figure 3C. Freezing in the vehicle group returned to high levels at the post-reactivation test, at which there was again no effect of 3HPG (Figure 4D; *F*_(1,14)_ = 0.36, *p* = 0.56). Therefore, infusion of 3HPG alone did not result in post-reactivation freezing impairments.

### Systemic ACEA + MK-801 impairs post-reactivation contextual freezing

**Figure 5:**
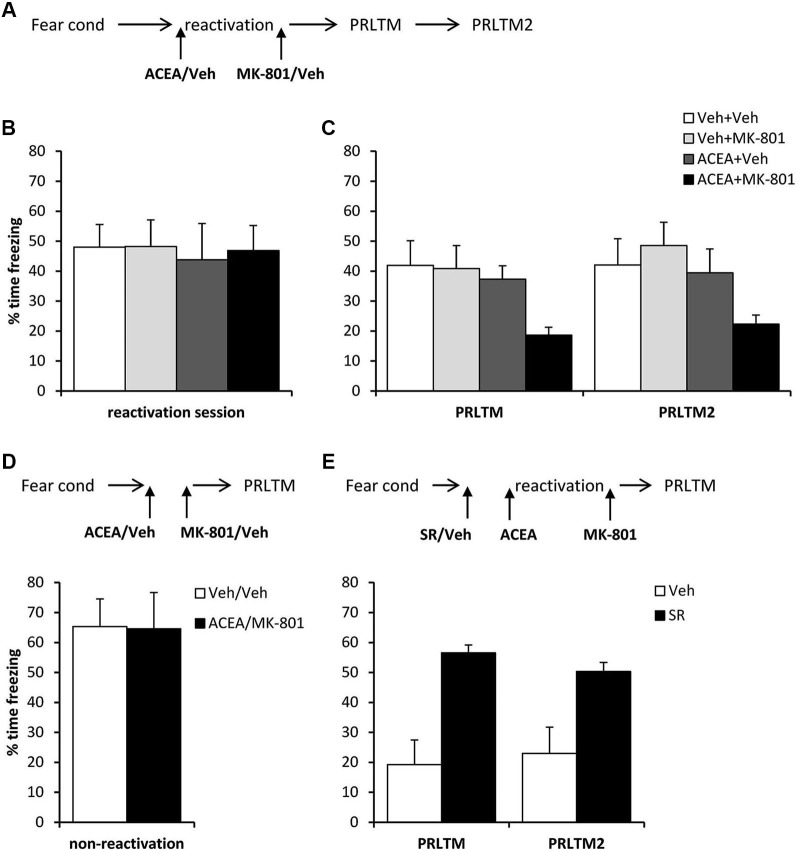
**Stimulation of memory destabilization by systemic ACEA injection**. (**A**)**** Schematic representation of the experimental procedure for panels B and C. (**B**) Prior to injection, there were no pre-existing differences in contextual freezing at memory reactivation. (**C**)**** Injection of ACEA 5 min prior to the reactivation session, combined with immediate post-reactivation MK-801injection impaired memory reconsolidation. In contrast, injection of ACEA or MK-801 alone had no amnestic effect. Contextual freezing was assessed 24 h (PRLTM) and 7 days (PRLTM2) after memory reactivation. (**D**) Injection of both ACEA and MK-801 in the absence of memory reactivation did not affect subsequent contextual freezing. (**E**) Pre-treatment with SR141716A prevented the amnestic effect of ACEA + MK-801 injection. Data presented as mean + s.e.m. (*N* = 8 per group).

After contextual fear conditioning, rats were injected with ACEA or vehicle prior to the reactivation session (Figure 5A), at which there were no differences between the groups in the levels of contextual freezing (Figure 5B; ACEA × MK-801 interaction: (*F*_(1,28)_ = 0.027, *p* = 0.87); main effect of ACEA: (*F*_(1,28)_ = 0.10, *p* = 0.75); main effect of MK-801: (*F*_(1,28)_ = 0.034, *p* = 0.85)). Immediately after the reactivation session, the rats were injected with MK-801 or vehicle. At the subsequent test sessions (Figure 5C), there was a significant effect of ACEA (*F*_(1,28)_ = 6.18, *p* = 0.019), but no ACEA × MK-801 (*F*_(1,28)_ = 1.86, *p* = 0.18) or session × ACEA × MK-801 (*F*_(1,28)_ = 0.27, *p* = 0.61) interactions. Planned comparisons in the rats that did not receive ACEA revealed no effect of MK-801 alone (main effect of MK-801: *F*_(1,14)_ = 0.078, *p* = 0.78; session × MK-801: *F*_(1,14)_ = 0.60, *p* = 0.45), but there was an effect of MK-801 in ACEA-injected groups (main effect of MK-801: *F*_(1,14)_ = 9.78, *p* = 0.007; session × MK-801: *F*_(1,14)_ = 0.052, *p* = 0.82). Moreover, there was no amnestic effect of ACEA alone (main effect of ACEA: *F*_(1,14)_ = 0.181, *p* = 0.68; session × ACEA: *F*_(1,14)_ = 0.036, *p* = 0.85). Thus the co-administration of ACEA and MK-801, but neither injection alone, impaired subsequent contextual freezing.

In order both to rule out the possibility that the combined injection of ACEA and MK-801 had non-specific effects on memory, and to confirm that the observed impairment was dependent upon memory reactivation, we injected ACEA and MK-801 at an interval of 7 min in the absence of memory reactivation (Figure 5D). Compared to vehicle control, ACEA and MK-801 did not impair subsequent contextual freezing (*F*_(1,14)_ = 0.002, *p* = 0.96). This demonstrated the reactivation-dependence of the ACEA–MK-801-mediated amnesia.

Furthermore, in order to confirm that the effect of ACEA + MK-801-mediated amnesia was critically dependent upon CB1 receptor activation, rather than some other non-specific effect of the dual drug administration, ACEA + MK-801-injected rats were pre-treated with the CB1 receptor antagonist SR141716A or vehicle control (Figure 5E). Compared to vehicle control, SR141716A-injected rats showed significantly higher levels of contextual freezing at both post-reactivation tests (main effect of SR141716A: *F*_(1,14)_ = 21.0, *p* < 0.001; session × SR141716A: *F*_(1,14)_ = 0.93, *p* = 0.35). This demonstrated that SR141716A protected against the amnestic effect of systemic ACEA–MK-801 injection, which is supported by the visual observation that SR141716A pretreatment had the impact of returning levels of contextual freezing at the post-reactivation tests to those observed in the control rats in Figure 5C.

## Discussion

The present results show that co-infusion of the cannabinoid CB1R agonist ACEA and the IKK inhibitor Sulf into the dorsal hippocampus immediately after contextual fear memory reactivation resulted in a subsequent impairment in contextual freezing 24 h and 7 days later. This was observed under conditions that failed to reveal any effect of ACEA or Sulf alone, and was both critically dependent upon the memory reactivation session and was replicated by systemic injections of ACEA and MK-801. Moreover, post-reactivation dorsal hippocampal ACEA infusion resulted in an upregulation of Zif268 protein that was not observed following memory reactivation alone. Finally, intra-hippocampal infusions of TTX but not the metabotropic glutamate agonist 3HPG impaired the amnestic effect of ACEA and Sulf, whereas both substances acutely impaired contextual fear memory expression.

The effect of combined ACEA + Sulf infusion and ACEA + MK-801 injection to impair contextual freezing is consistent with a disruption of contextual fear memory reconsolidation. The reactivation-dependence and long-lasting nature of the amnesia are characteristic features of reconsolidation deficits (Dudai, 2004; Nader and Hardt, 2009). Sulf and MK-801 have each been demonstrated to impair contextual fear memory reconsolidation (Lubin and Sweatt, 2007; Charlier and Tirelli, 2011; Lee and Hynds, 2013), but under the present conditions failed to do so when administered alone. This is likely because the 2-min memory reactivation session was insufficient to destabilize the strongly-conditioned fear memory. Indeed, with a similar conditioning session to that employed here, a 10-min, but not 3-min, reactivation session was able to destabilize the retrieved memory (Suzuki et al., 2004). Therefore, the present behavioral parameters fall outside the “boundary conditions” (Nader and Hardt, 2009) of contextual fear memory reconsolidation. Nevertheless, the co-infusion of ACEA and Sulf did disrupt memory reconsolidation under these conditions. Given that dorsal hippocampal CB1R activation is critical for contextual fear memory destabilization (Suzuki et al., 2008), our interpretation is that the ACEA-mediated stimulation of CB1R activity potentiated the destabilization process, thereby necessitating an NMDAR and IKK-dependent reconsolidation process that was disrupted by MK-801 or Sulf. This interpretation is strengthened by the effect of the selective CB1R antagonist SR141716A to prevent the amnestic effect of ACEA + MK-801. SR141716A has previously been used at the present dose to demonstrate that cannabidiol enhances contextual fear memory extinction via CB1Rs (Bitencourt et al., 2008). Here, the lack of reconsolidation impairment in rats pre-treated with SR141716A strongly suggests that the amnestic effect of ACEA + MK-801 is mediated by the activation of CB1Rs by ACEA. Therefore, this indicates that the administration of MK-801 does not alter the predicted pharmacological action of ACEA at CB1Rs. However, we cannot similarly rule out completely the possibility that pre-treatment with ACEA has some direct modulatory effect on the pharmacological action of MK-801. Indeed, this is an interpretative issue with all studies of memory destabilization, which require the administration of two drugs in close temporal proximity (Lee et al., 2008; Suzuki et al., 2008; Milton et al., 2013). Nevertheless, regardless of the precise mechanism of action of MK-801 in the present study, the interpretation remains that CB1R activation stimulates the destabilization of contextual fear memories. Moreover, given the common effects of systemic and intra-hippocampal ACEA upon contextual freezing, a primary locus of action of systemically-administered ACEA is likely to be the dorsal hippocampus. Thus ACEA enhanced hippocampal contextual fear memory destabilization under memory reactivation conditions that were sub-optimal for memory destabilization.

ACEA infusion alone did not have a disruptive effect upon the reactivated contextual fear memory. This suggests that CB1R activation allows memory destabilization, but does not have any deleterious consequences for memory reconsolidation. There are, however, reports of the CB1R agonist WIN55212-2 impairing memory reconsolidation when infused into the amygdala or insular cortex in a fear-potentiated startle (Lin et al., 2006) and conditioned taste aversion (Kobilo et al., 2007) setting, respectively. The reconsolidation-disrupting effects of WIN55212-2 might be explained by the differential neural loci, especially as the cellular mechanisms of destabilization and reconsolidation appear to differ between the amygdala and hippocampus (Duvarci et al., 2005; Ben Mamou et al., 2006; Lee and Hynds, 2013; Milton et al., 2013). Infusion of anandamide into the dorsal hippocampus did result in a moderate, but significant, impairment of memory reconsolidation (de Oliveira Alvares et al., 2008). Therefore, it remains unclear why ACEA and anandamide have seemingly different effects on the reconsolidation of contextual fear memories. Importantly, it should be recognized that reconsolidation impairments are not easily distinguishable from extinction enhancements (Lattal and Wood, 2013), and CB1R agonism has also been shown to have effects interpreted as a potentiation of extinction memory (Pamplona et al., 2006; de Oliveira Alvares et al., 2008), consistent with the effect of CB1R antagonism to impair extinction memory (Suzuki et al., 2004). Therefore, it also remains unclear what the mechanism of action is for the memory-impairing effects of CB1R agonists.

In the present study, ACEA was infused into the hippocampus immediately after memory reactivation. While the first study of memory destabilization showed that the NR2B NMDA receptor antagonist ifenprodil only impaired memory destabilization when it was infused into the amygdala prior to memory reactivation (Ben Mamou et al., 2006), both the CB1R antagonist SR141716A and the L-type voltage-gated calcium channel blocker verapamil successfully impaired contextual fear memory destabilization when infused into the dorsal hippocampus immediately after memory reactivation (Suzuki et al., 2008). This indicates that, at least in the hippocampus, the process of memory destabilization outlasts the reactivation session itself.

It has previously been established that memory expression is not necessary for memory destabilization (Ben Mamou et al., 2006; Forcato et al., 2011; Rodriguez-Ortiz et al., 2012; Milton et al., 2013). In the present study, we showed similar results using the metabotropic glutamate receptor 1 agonist 3HPG. 3HPG acutely impaired the expression of contextual freezing, similar to previously-reported effects (Szapiro et al., 2001). However, it did not affect the amnestic impact of ACEA and Sulf. Nor did 3HPG impair reconsolidation by itself. Given that reconsolidation impairments constitute reactivation-dependent amnestic effects, memory reactivation, if not expression, is critically important for memory destabilization. The reactivation of a memory ultimately depends upon neuronal activity in the mnemonic locus, and here we disrupted such neuronal activity using the sodium channel blocker TTX. TTX infusion into the dorsal hippocampus led to two effects. First, it acutely impaired contextual fear memory expression, consistent with previous observations (Lorenzini et al., 1996) and the necessity for a memory to be reactivated in order to be expressed. Second, TTX prevented memory destabilization. Infusion of TTX protected against the amnestic effect of ACEA + Sulf. This protection against reactivation-dependent amnesia is characteristic of disruptions of memory destabilization (Ben Mamou et al., 2006; Lee, 2008; Lee et al., 2008; Suzuki et al., 2008; Milton et al., 2013). Therefore, activity-dependent memory reactivation underpins, in parallel, both memory expression and memory destabilization.

The processes that result from a memory reminder have been highlighted by a recent study by Barreiro et al. (2013). They note that a reactivated memory can undergo different fates, including expression and “labilization/reconsolidation” (here, destabilization-reconsolidation). Here, we expand upon this framework to add that the process of a reminder leading to an active memory is, by definition, memory reactivation (Lewis, 1979) and is dependent upon neuronal activity. We confirm that for rodent contextual fear memories, memory expression is doubly dissociable with memory destabilization, the latter process triggering subsequent reconsolidation in order to return the memory to a stable inactive state. Given the ambiguity of the definition of memory “retrieval”, being simultaneously conflated with memory reactivation and expression as defined above, we would propose that descriptions of reminder-induced memory processing avoid the term altogether, instead focussing on the more tractable concepts of reactivation, expression, destabilization and reconsolidation.

In summary, the present results show that contextual fear memory destabilization can be robustly enhanced by the cannabinoid CB1R agonist ACEA. This was observed in three separate experiments and raises the potential for pharmacologically enhancing the destabilization of problematic memories in reconsolidation-based treatment approaches for posttraumatic stress disorder (Debiec and Altemus, 2006) and drug addiction (Milton and Everitt, 2010). Moreover, while memory destabilization and memory expression are dissociable processes, both are commonly dependent upon memory reactivation-induced dorsal hippocampal neuronal activity. This accounts, therefore, for the behavioral impact of memory reactivation being separable from the process and function of memory reconsolidation (Gisquet-Verrier and Riccio, 2012). Whether or not the former is dependent upon the expression of a memory remains to be determined.

## Conflict of interest statement

The authors declare that the research was conducted in the absence of any commercial or financial relationships that could be construed as a potential conflict of interest.
